# Supplementary figures and images for: Vaccinia virus-based vaccines confer protective immunity against SARS-CoV-2 virus in Syrian hamsters
Source: PLoS One. 2021 Sep 9;16(9):e0257191. doi: 10.1371/journal.pone.0257191 (PMC8428573; doi:10.1371/journal.pone.0257191)

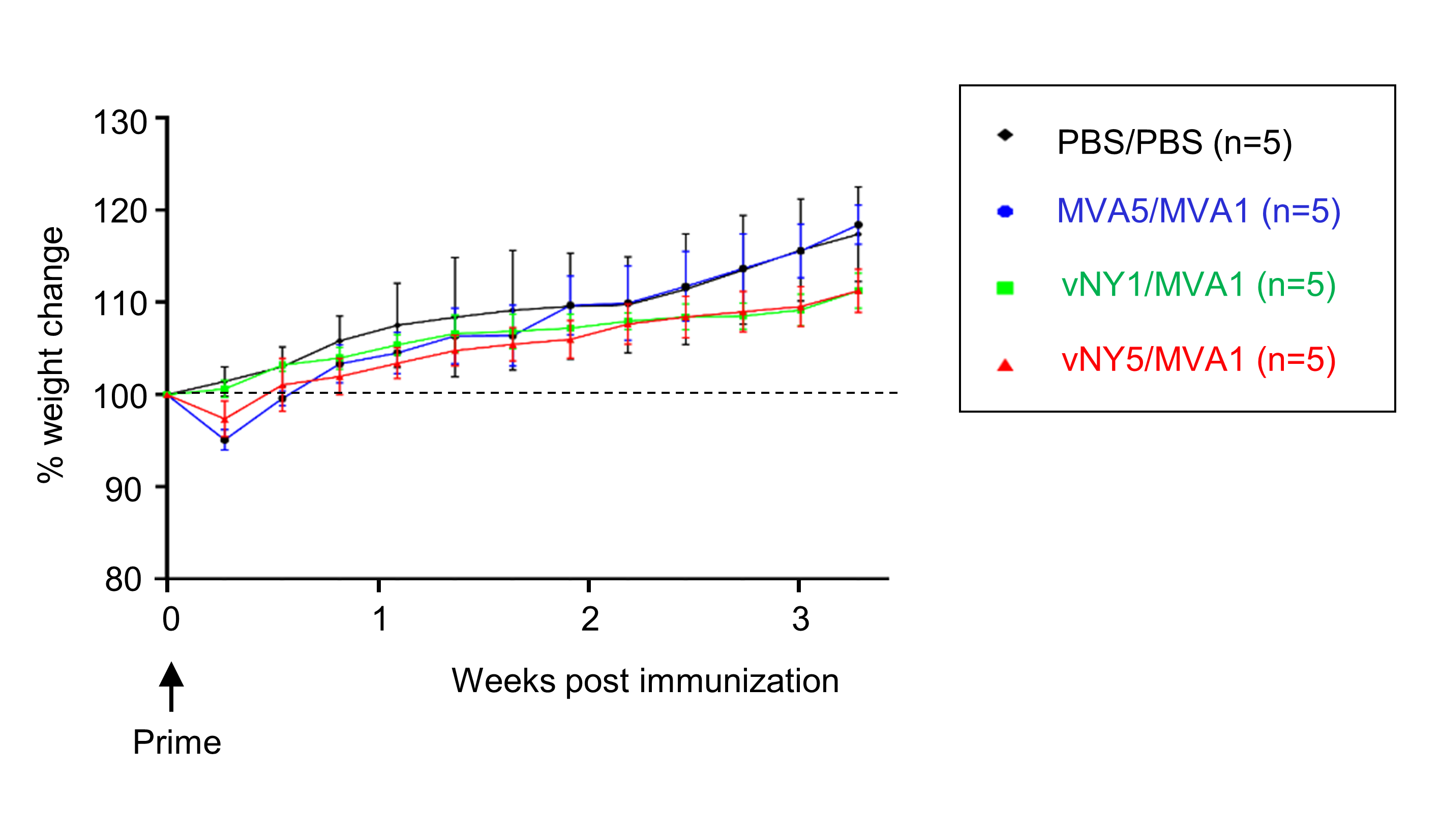

Supplement: S1 Fig — (TIF) [file pone.0257191.s001.tif]

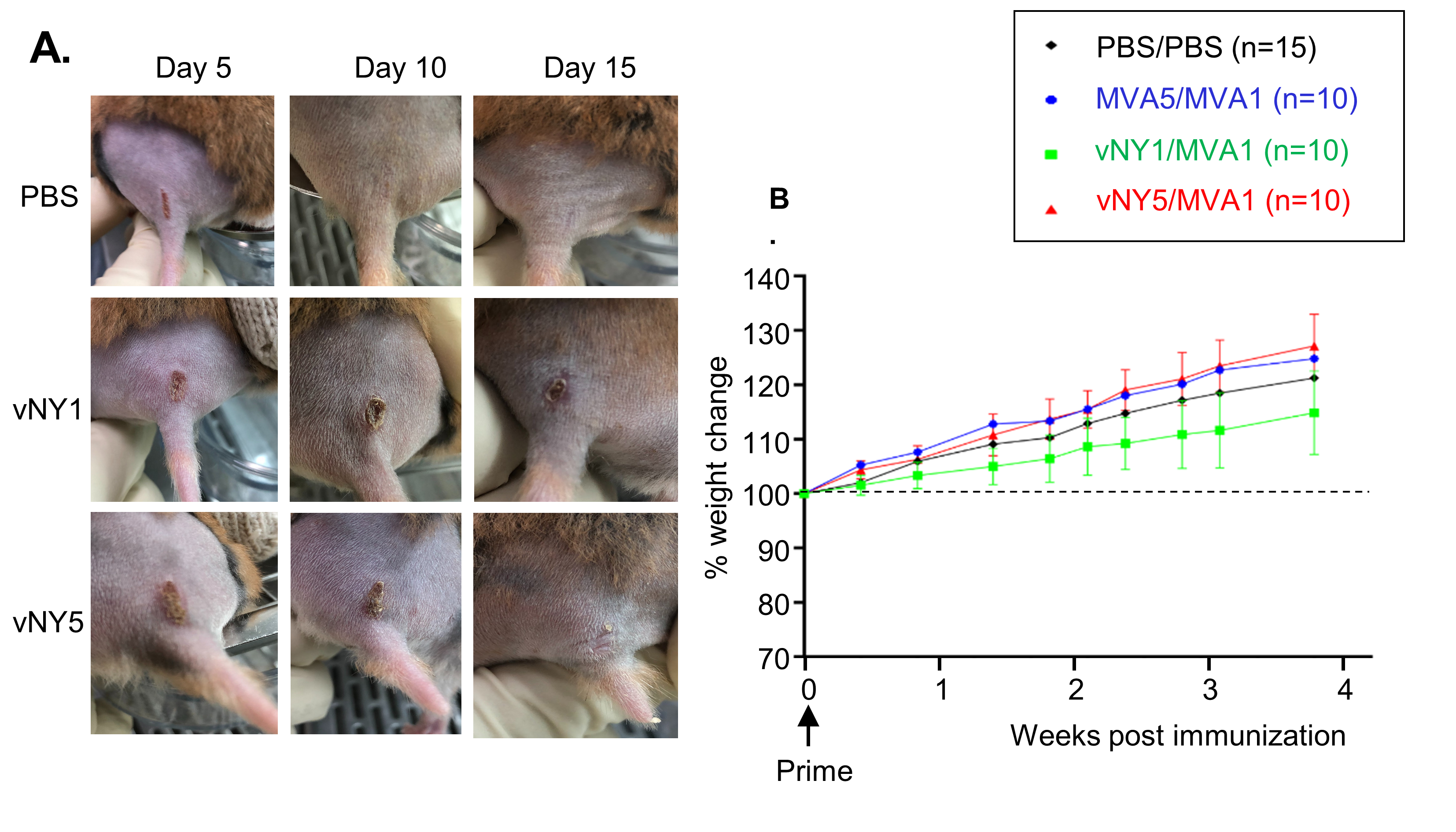

Supplement: S2 Fig — (A). Images of skin scarification in Syrian hamsters at days 5,10 and 15 after primary immunization. (B) Weight change in Syrian hamsters after immunization with one of the three regimens. (TIF) [file pone.0257191.s002.tif]

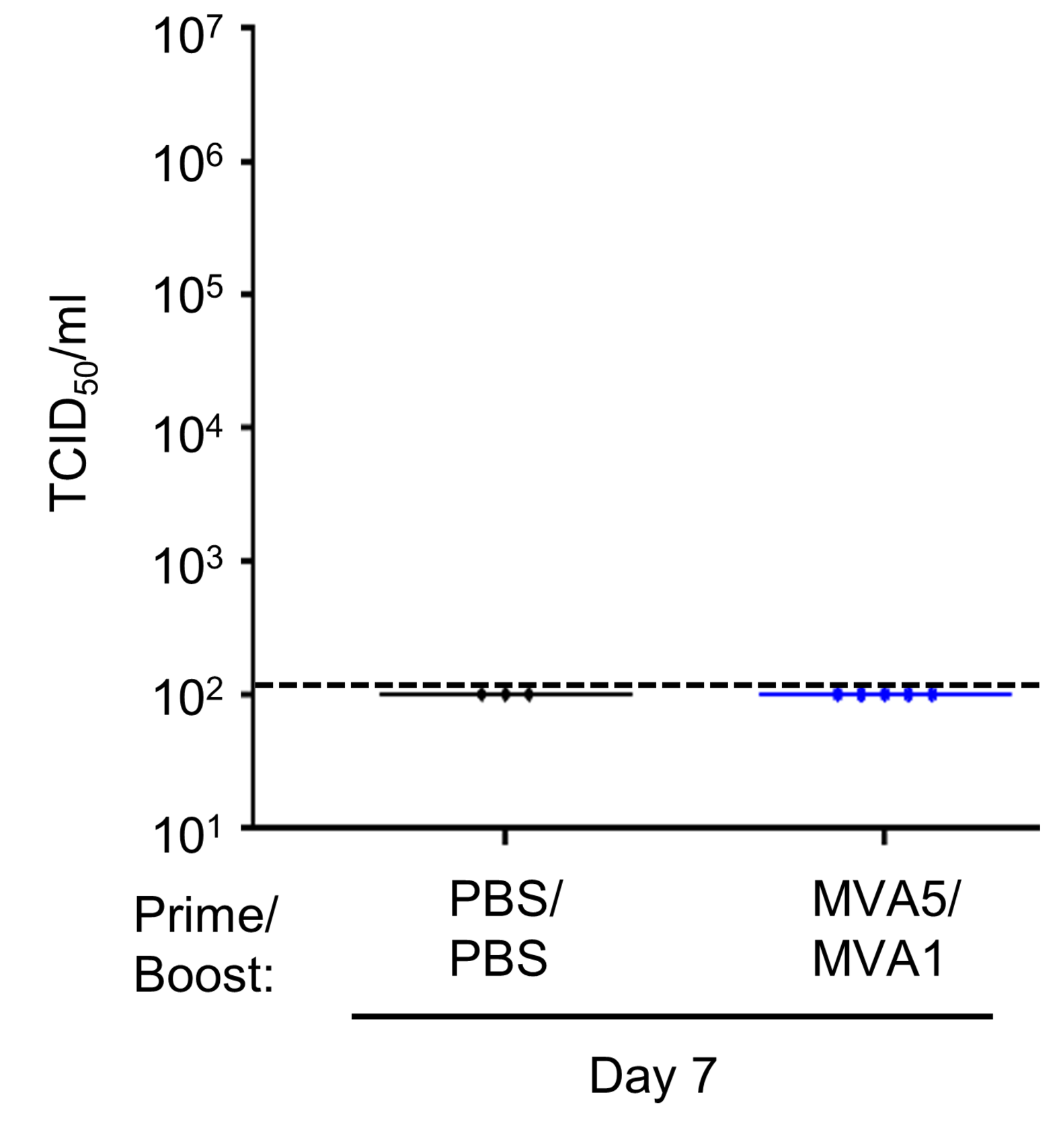

Supplement: S3 Fig — (TIF) [file pone.0257191.s003.tif]
