# Supplementary material for: Vaccinia virus-based vaccines confer protective immunity against SARS-CoV-2 virus in Syrian hamsters
Source: PLoS One. 2021 Sep 9;16(9):e0257191. doi: 10.1371/journal.pone.0257191 (PMC8428573; doi:10.1371/journal.pone.0257191)
Supplement: S1 Raw images — (PDF) [file pone.0257191.s004.pdf]

S_1_raw images

Immunoblot images were taken on the instrument UVP Biospectrum 815 using Visionworks software (8.20.1709.9551).

Fig. 1D

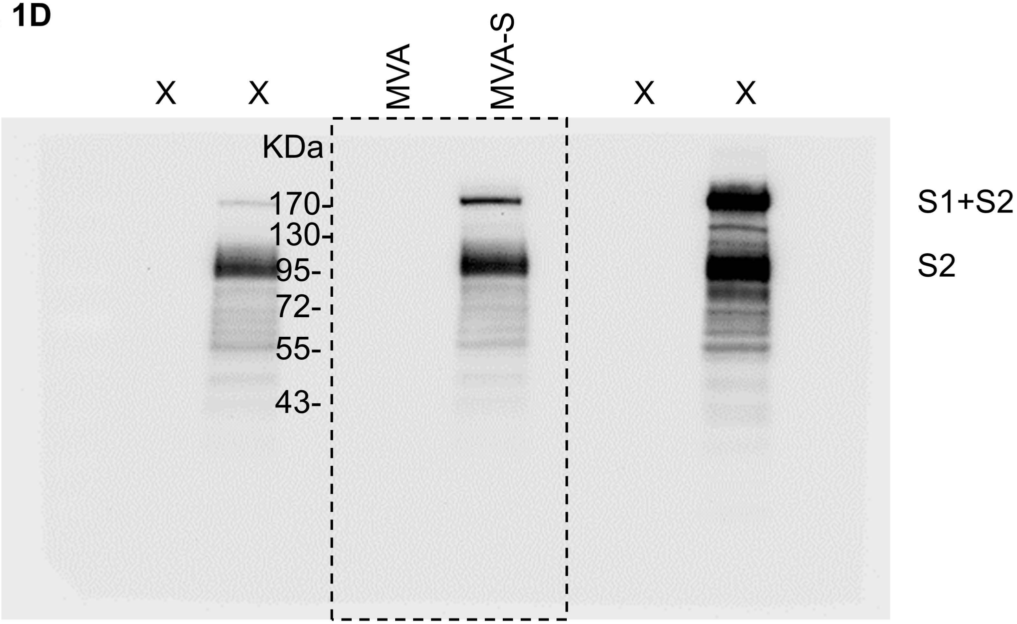

Fig. 1D

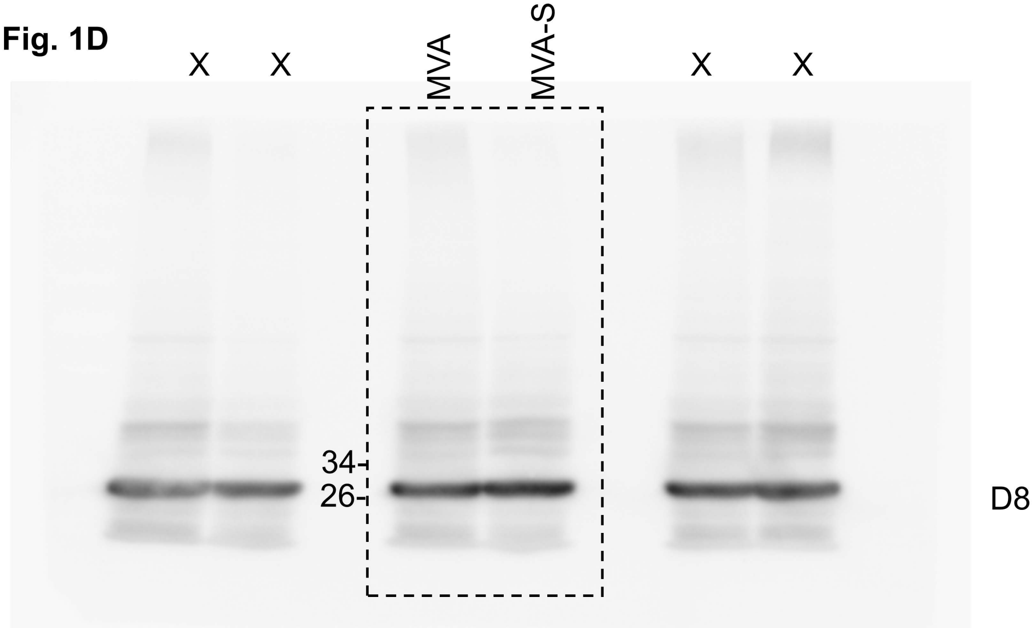

Fig. 1D

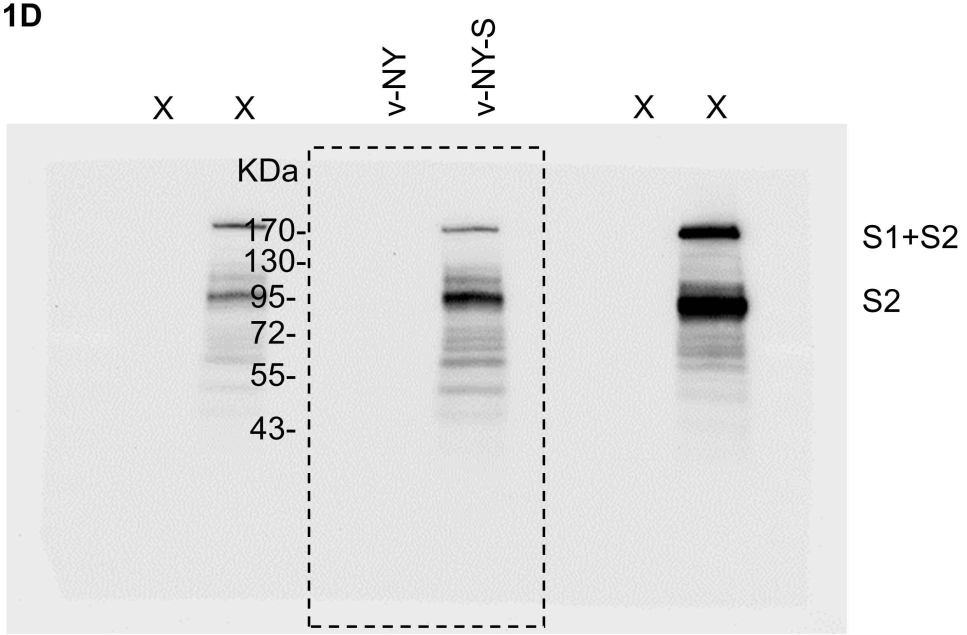
X
X
v-NY
v-NY-S
X
X
KDa
170-
130-
95-
72-
55-
43-
S1+S2
S2

Fig. 1D

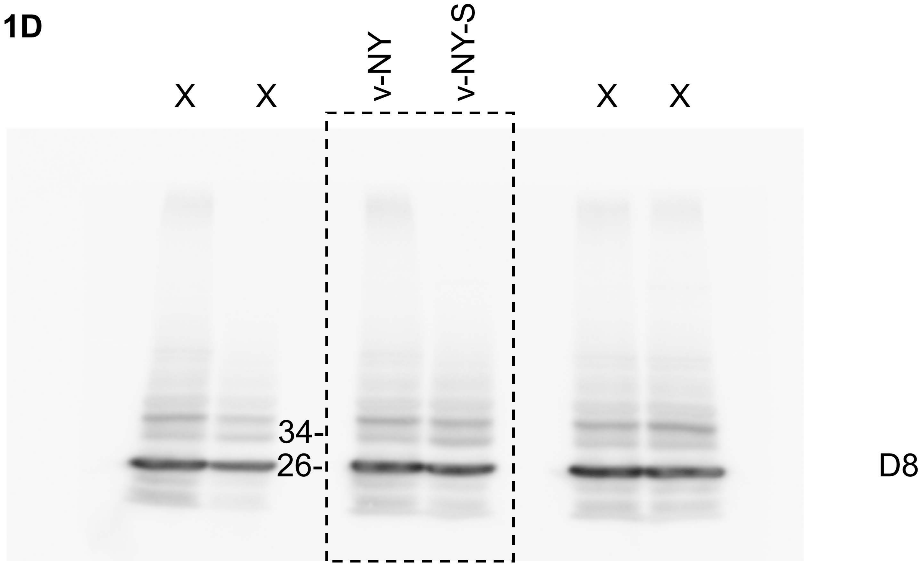
X
X
v-NY
v-NY-S
X
X
34-
26-
D8

Note: Immunoblot images in Fig. 2D and Fig.4D were probed in small individual stripes for each serum sample and the full image of each strip is shown below.

Fig 2D.

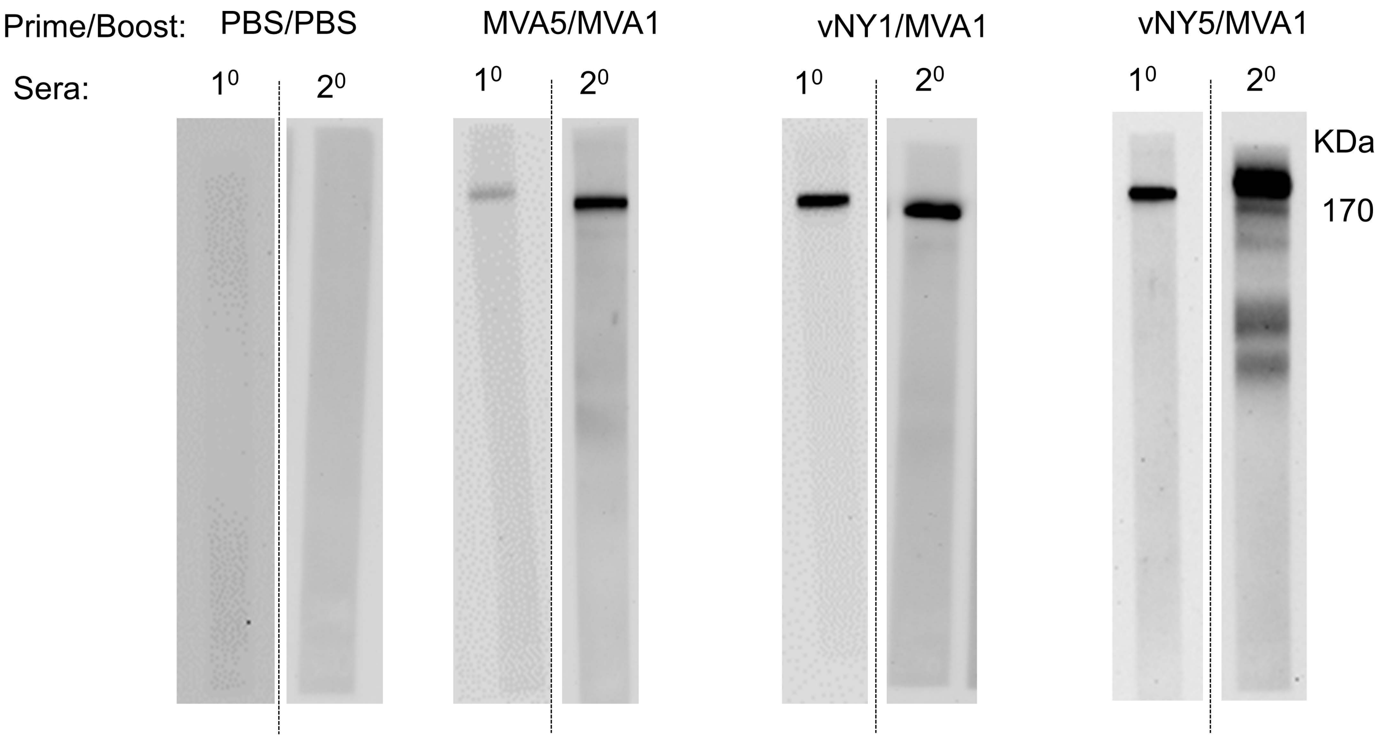

Fig 4D.

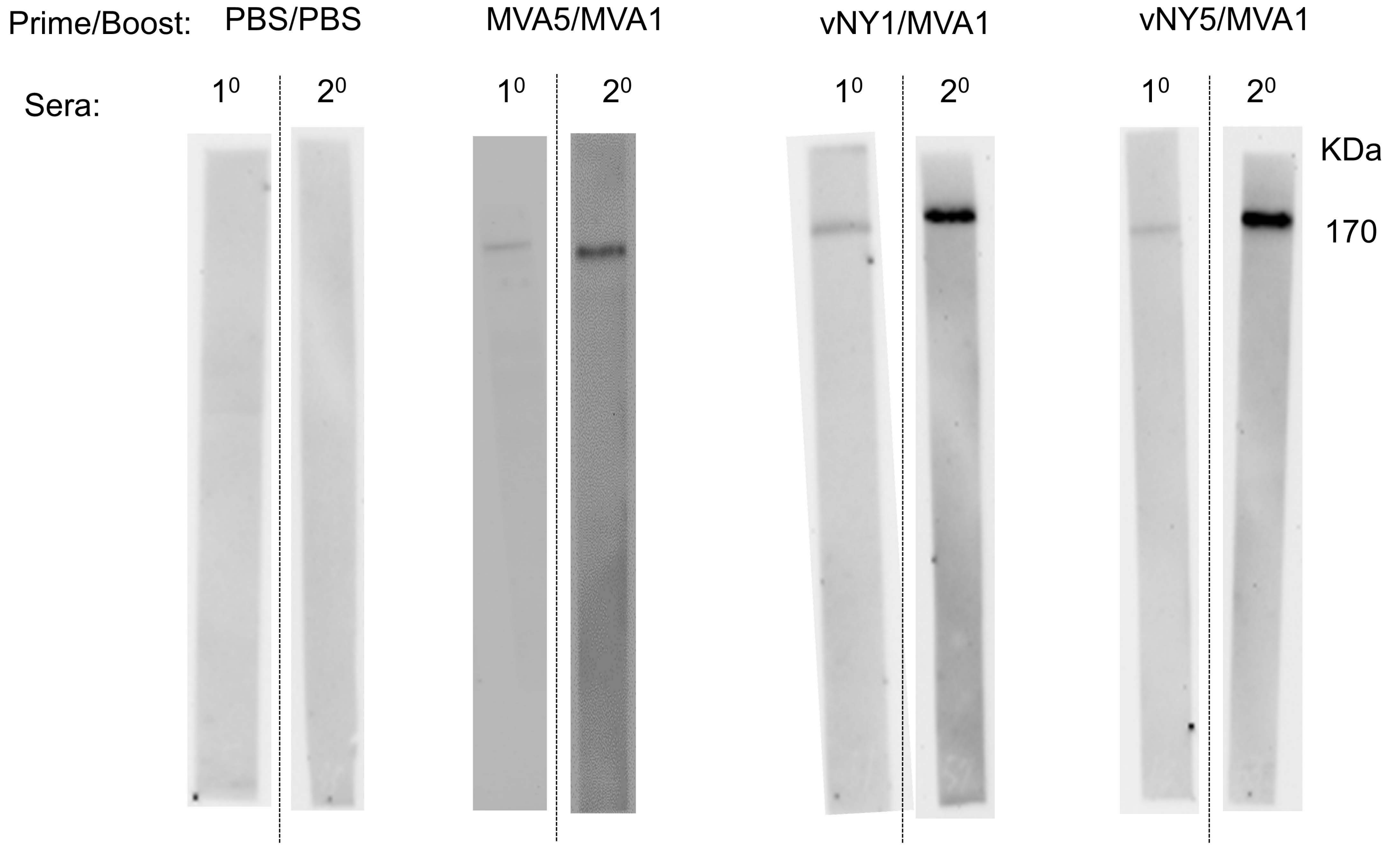
Prime/Boost:
PBS/PBS
MVA5/MVA1
vNY1/MVA1
vNY5/MVA1
Sera:
1^{0}
2^{0}
1^{0}
2^{0}
1^{0}
2^{0}
1^{0}
2^{0}
KDa
170
